# Analysis of protein expression profiles in the thymus of chickens infected with Marek’s disease virus

**DOI:** 10.1186/1743-422X-9-256

**Published:** 2012-11-01

**Authors:** Xuming Hu, Aijian Qin, Kun Qian, Hongxia Shao, Chuan Yu, Wencai Xu, Ji Miao

**Affiliations:** 1 Ministry of Education Key Lab for Avian Preventive Medicine, Yangzhou University, No.12 East Wenhui Road, Yangzhou, Jiangsu 225009, P.R.China; 2 Key Laboratory of Jiangsu Preventive Veterinary Medicine, Yangzhou University, Yangzhou, 225009, P.R. China

## Abstract

**Background:**

Marek’s disease virus (MDV) is a highly cell-associated oncogenic α-herpesvirus that causes a disease characterised by T-cell lymphomas. The pathogenesis, or the nature of the interaction of the virus and the host, in the thymus are still unclear.

**Results:**

In this study, we identified 119 differentially expressed proteins using two-dimensional electrophoresis and mass spectrometry from the thymuses of chickens infected with the RB1B strain of MDV. These differentially expressed proteins were found mainly at 21, 28 and 35 days post-infection. More than 20 of the differentially expressed proteins were directly associated with immunity, apoptosis, tumour development and viral infection and replication. Five of these proteins, ANXA1, MIF, NPM1, OP18 and VIM, were further confirmed using real-time PCR. The functional associations and roles in oncogenesis of these proteins are discussed.

**Conclusions:**

This work provides a proteomic profiling of host responses to MDV in the thymus of chickens and further characterises proteins related to the mechanisms of MDV oncogenesis and pathogenesis.

## Background

Marek’s disease virus (MDV) is a highly cell-associated oncogenic α-herpesvirus that leads to serious economic losses in the poultry industry [1,2]. Marek’s disease (MD) is a lymphoproliferative disease characterised by immunosuppression, neurological disorders, and neoplastic T-cell lymphomas in chickens. MD was the first tumour disease to be prevented by vaccination, and thus, provides an important animal model for the study of viral cancer development and immunity [3].

The primary target cells for MDV infection in the chicken *in vivo* are B cells, then T cells, and eventually the formation of a T-cell lymphoma occurs [4]. The mechanism of lymphoma formation is very complex and has not yet been clarified. In recent years, the dynamics of host-protein expression in chicken immune organs have been studied at different phases of MDV infection using two-dimensional polyacrylamide gel electrophoresis (2-DE) followed by the identification and characterisation of the proteins by mass spectrometry (MS) [5-7]. In the spleens of MDV-infected chickens, 61 protein spots representing 48 host proteins have been detected. These proteins are involved in a variety of cellular processes, including antigen processing and presentation, ubiquitin-proteasome protein degradation (UPP), formation of the cytoskeleton, cellular metabolism, signal transduction, and translation regulation [5]. In the bursa of Fabricius, 24 differentially expressed proteins associated mainly with tumour biology, protein folding, signal transduction, immunology, cell proliferation and apoptosis have been successfully identified, and the tumour-associated proteins were significantly increased at 14 and 21 days post-infection (dpi) [6]. Furthermore, 20 proteins have been found to be differentially expressed in the spleen when comparing MD-susceptible B19 and MD-resistant B21 chickens [7]. These studies have characterised the proteomic profiles of the host response to MDV in chickens and are the basis for illustrating the mechanism of MD lymphoma formation. In addition, Niikura et al. found that a lytic infection with MDV up-regulates the cell surface expression of MHC class II infected cells [8] but down-regulates the expression of MHC class II cells in the spleens of MDV-infected chickens [9].

The thymus is the specific organ in which the maturation and differentiation of avian T lymphocytes take place. T-cell immune suppression and lymphomas due to MDV infection and latent infections are predominantly related to activated CD4+ T lymphocytes. Morimura et al. reported that MDV can induce apoptosis and the down-regulation of CD8 molecules on peripheral CD4+ T cells and the thymus, which could contribute to immune suppression [10-12]. However, all the changes in the protein profiles that occur after MDV infection have not been reported. Such changes may indicate the nature of the interaction between MDV and host and provide some clues to the pathogenesis of MDV. In this study, 119 proteins that were differentially expressed in the thymus in response to MDV infection were identified by two-dimensional electrophoresis and mass spectrometry techniques at 4, 7, 14, 21, 28, 35 and 42 dpi. These proteins are associated with a wide range of biological processes, including metabolism, immunity, apoptosis, death, tumour development and virus infection and replication. These proteins provide some information for further interpretations of the pathogenesis and oncogenesis of MDV.

## Results

### Pathological changes associated with MDV infection at different stages

**Figure 1:**
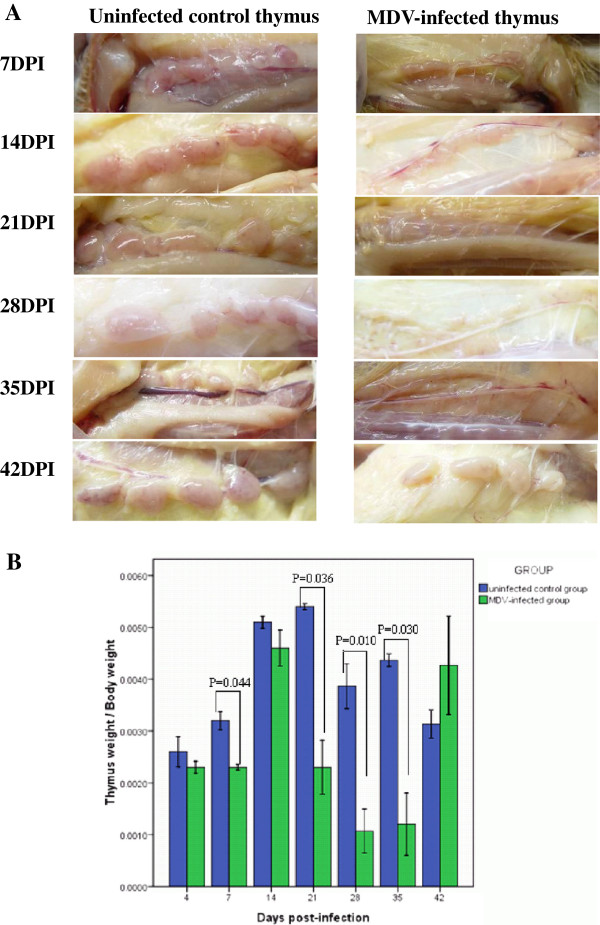
**(A) The macroscopic appearance of the thymus at different stages of infection and in the control group.** (**B**) The differences in the thymus and body weight between the two groups at the different stages of infection.

The thymus of each chicken displayed severe atrophy at 21, 28 and 35 dpi with RB1B, and the size of the thymus gradually returned to normal at 42 dpi (Figure 1A). Compared with the control group, the thymus and body weight parameters of the MDV-infected chickens displayed significant differences at 7, 21, 28 and 35 dpi (Figure 1B).

### Differential expression in the MDV-infected chicken thymuses at different time points

**Figure 2:**
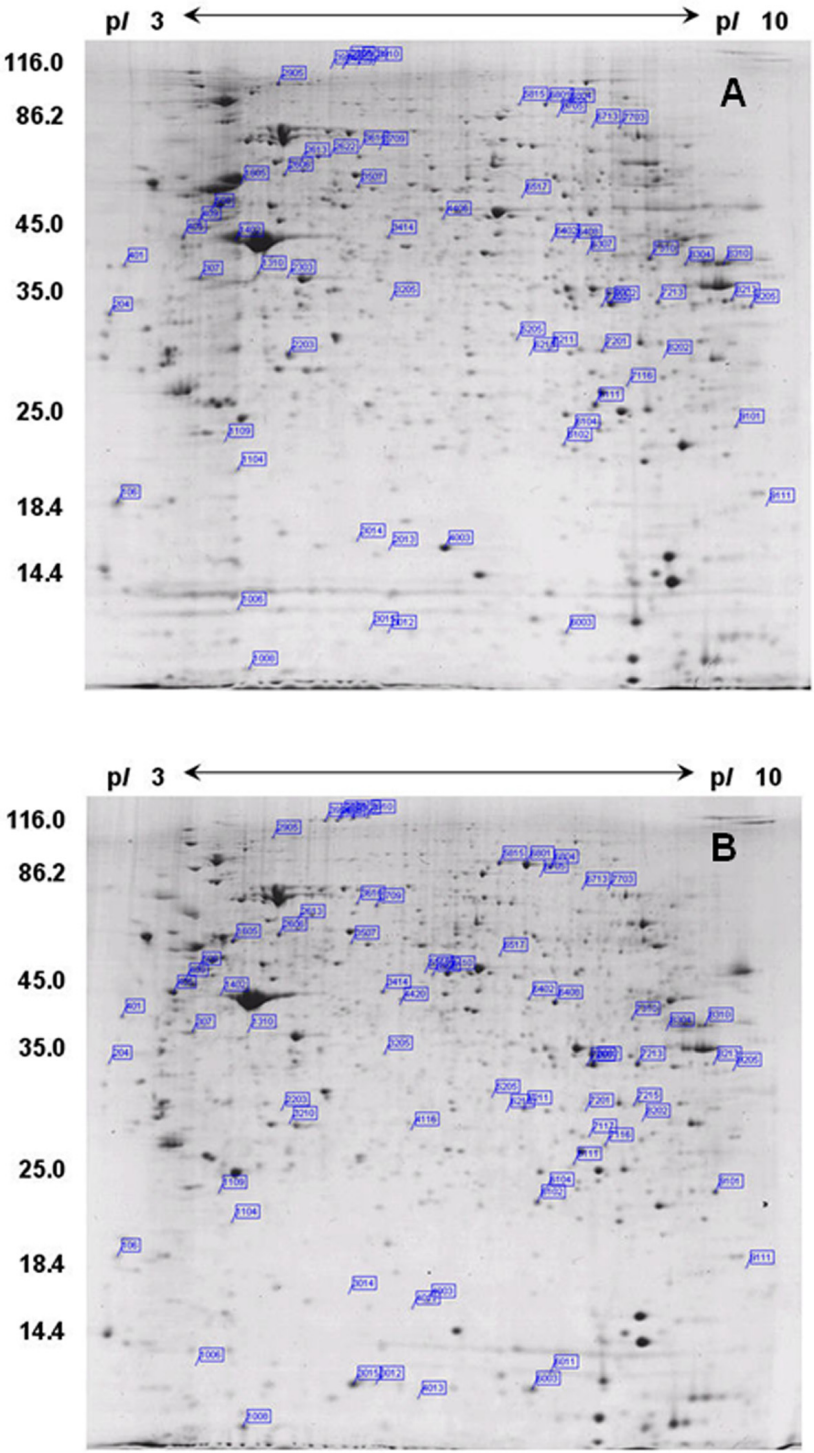
**The analysis of the chicken thymus proteins by two-dimensional gel electrophoresis.** Above images represent the proteome of chicken thymus at 21 dpi. The differentially expressed proteins (p ≤ 0.05 and fold change ≥ 2) are marked on the gel. **A**. Uninfected control thymus. **B**. MDV-infected thymus.

**Figure 3:**
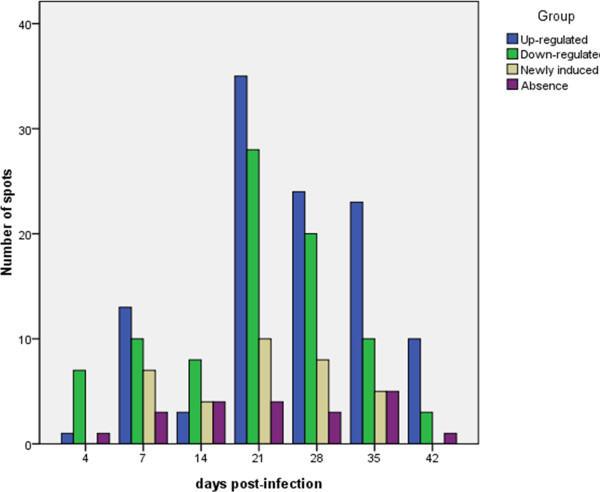
**Comparison of the total numbers of protein spots that were significantly differentially expressed in the MDV-infected thymus at the different time points.** A total of 250 protein spots were detected, among which 9, 33, 19, 77, 55, 43 and 14 were detected at 4, 7, 14, 21, 28, 35 and 42 dpi, respectively. Notably, some of the protein spots were detected at least once during the different sampling points, and thus, only 119 protein identities required MS analyses.

To obtain protein expression profiles at different time points in the thymus from the MDV-infected and uninfected control chickens, total protein was collected from the thymus specimens, separated by two-dimensional electrophoresis and analysed using PDQuest 8.0.1 software. More than 1000 protein spots could be detected in each gel (Figure 2). In total, 250 protein spots were detected as either quantitatively (p≤0.05 and fold change≥2) or qualitatively differentially expressed (35 that were novel and 20 that were no longer visible) in the thymus.These spots were predominantly identified at 21, 28 and 35 dpi, which was consistent with the pathological observations (Figure 3).

### MS analysis and GO annotations

Some protein spots were detected more than once differentially expressed during experiment, resulting less number of identities than the total number of spots. In total, 119 differentially expressed proteins were successfully identified by MALDI-TOF/TOF (Table 1). These proteins are involved in a wide range of biological processes, including metabolism, immunity, apoptosis, death, tumour development, virus infection and replication. More than 20 of the differentially expressed proteins were directly associated with immunity, apoptosis, tumour development and viral infection and replication, including macrophage migration inhibitory factor (MIF), heat shock protein 90 alpha (Hsp90alpha), and annexin-A1 (Anx-A1).

**Table 1:** Fundamental information of 119 differentially expressed protein spots identified by MS

**SSP**	**Protein Name**	**Fold change in expression**							**Accession number**	**MW**	**PI**
		**4dpi**	**7dpi**	**14dpi**	**21dpi**	**28dpi**	**35dpi**	**42dpi**			
**Actin cytoskeleton and cellular structural proteins**											
**3908**	**collagen alpha-1(VI) chain precursor**	**−1.05**	**1.57**	**1.44**	4.53**	3.47**	**1.14**	**−1.65**	**gi|49225581**	**107916.8**	**5.63**
**3910**	**collagen alpha-1(VI) chain precursor**	**1.32**	2.92**	**−1.66**	3.77**	3.72*	**−1.73**	**1.06**	**gi|49225581**	**107916.8**	**5.63**
**3914**	**collagen alpha-2(VI) chain precursor**	**1.01**	**1.79**	**1.54**	4.11**	**−1.2**	**1.35**	**−1.01**	**gi|45384382**	**109108.4**	**5.66**
**3915**	**collagen alpha-2(VI) chain precursor**	**−1.33**	2.24**	**1.4**	3.58**	**1.71**	**1.89**	**1.09**	**gi|45384382**	**109108.4**	**5.66**
**508**	**keratin, type I cytoskeletal 15**	**−1.22**	**−1.17**	**1.06**	2.67**	**1.28**	**−1.02**	**−1.03**	**gi|47604932**	**48060.8**	**5.11**
**6713**	**keratin, type II cytoskeletal 1**	**−1.03**	**−1.68**	**1.23**	−2.25**	**−1.43**	**−1.2**	**−1.69**	**gi|119395750**	**65999**	**8.15**
**4502**	**keratin, type II cytoskeletal cochleal**	**1.69**	**−1.15**	−2.55**	**−1.2**	**1.64**	**1.42**	**−1.23**	**gi|45384378**	**53770.5**	**5.97**
**106**	**Chain B, Refined 1.8 Angstroms Resolution Crystal Structure Of Porcine Epsilon-Trypsin**	**1.34**	**−1.31**	**−1.14**	−2.62**	−2.28**	−2.63**	**−1.29**	**gi|999627**	**8813.5**	**6.67**
**9222**	**PREDICTED: LOW QUALITY PROTEIN: keratin, type II cytoskeletal 1-like**	**AB**	**IN**	**AB**	**AB**	**AB**	**AB**	**AB**	**gi|296211766**	**66379**	**8.16**
**6206**	**REDICTED: similar to Chain D, Crystal Structure Of Arp23 COMPLEX**	**1.08**	**1.34**	**1.07**	**−1.04**	**−1.08**	**1.21**	2.14**	**gi|118093746**	**34379.6**	**6.84**
**6211**	**PREDICTED: similar to Chain D, Crystal Structure Of Arp23 COMPLEX**	−2.11**	3.72**	**1.09**	4.76**	2.22**	4.28**	**1.73**	**gi|118093746**	**34379.6**	**6.84**
**314**	**beta-tropomyosin**	**−1.19**	**1.04**	**1.37**	**1.82**	**1.66**	2.86**	**1.43**	**gi|212809**	**32779.7**	**4.77**
**6601**	**fibrinogen beta chain precursor**	**−1.37**	**1.28**	**−1.01**	**1.88**	2.33**	**−1.2**	**−1.44**	**gi|267844833**	**54545.7**	**7.84**
**2606**	**plastin-2**	**−1.65**	2.04**	**1.23**	2.44**	2.24*	2.13**	**1.27**	**gi|56605886**	**69704.5**	**5.16**
**2206**	**beta-actin**	**−1.1**	**1.87**	**1.08**	**1.06**	**1.53**	2.08**	2.2**	**gi|154818367**	**41812.8**	**5.32**
**3906**	**collagen alpha-1(VI) chain precursor**	**−1.07**	**1.67**	**1.51**	4.29**	**1.68**	**1.55**	**−1.61**	**gi|49225581**	**107916.8**	**5.63**
**Enzymes**											
**4406**	**adenylosuccinate synthetase isozyme 2**	**−1.29**	**1.1**	**−1.39**	2.19*	2.2*	**−1.46**	**−1.16**	**gi|71895783**	**49423.4**	**5.93**
**4410**	**argininosuccinate synthase**	**1.48**	**1.56**	**1.49**	**IN**	**3.99**	2.64**	**−1.2**	**gi|61657937**	**46873.8**	**6.1**
**5402**	**argininosuccinate synthase**	**−1.63**	4.04**	**1.03**	**1.15**	**1**	**1**	**−1.45**	**gi|61657937**	**46873.8**	**6.1**
**5904**	**argininosuccinate synthase**	**1.35**	2.49**	**1.22**	**1.82**	**1.83**	**−1.23**	**−1.67**	**gi|61657937**	**46873.8**	**6.1**
**6705**	**ATP-dependent RNA helicase DDX3X**	**−1.03**	**−1.33**	**1.15**	−2.2**	−2.27**	**1**	**−1.55**	**gi|71895253**	**72004.5**	**6.54**
**3414**	**B-creatine kinase**	**−1.32**	**1.73**	**−1.3**	2.09**	**1.76**	**1.34**	**−1.08**	**gi|211235**	**42240.4**	**5.78**
**7213**	**L-lactate dehydrogenase A chain**	**−1.08**	**1.71**	**−1.03**	2.36**	2.63**	2.43**	**−1.4**	**gi|45384208**	**36491.2**	**7.75**
**6606**	**phosphoglucomutase 2**	**AB**	**REP**	**REP**	**−1.36**	**AB**	**−1.4**	**1.29**	**gi|71897287**	**67900.1**	**6.56**
**9221**	**PREDICTED: similar to 2,4-dienoyl-CoA reductase**	**AB**	**IN**	**AB**	**AB**	**AB**	**AB**	**AB**	**gi|50731694**	**35723.5**	**9.46**
**3205**	**PREDICTED: similar to pyridoxal kinase**	**AB**	**1.56**	**1.45**	2.74*	2.02**	2.45**	**−1.04**	**gi|118083963**	**43075.8**	**8.36**
**3207**	**RecName: Full=3-mercaptopyruvate sulfurtransferase; Short=MST**	**AB**	**AB**	**IN**	**AB**	**AB**	**AB**	**AB**	**gi|90110410**	**33002.5**	**6.11**
**6107**	**RecName: Full=Carbonic anhydrase 2; AltName:**	**−1.13**	**−1.45**	**1.13**	**1.38**	**3.04**	**1.19**	2.22*	**gi|115454**	**29388.4**	**6.56**
	**Full=Carbonate dehydratase II; AltName: Full=Carboni anhydrase II; Short=CA-II**										
**2622**	**PREDICTED: similar to serine/threonine kinase isoform 1**	**AB**	**1.17**	**1.44**	**REP**	**−1.22**	**−1.43**	−2.46**	**gi|118094908**	**57883.8**	**6.04**
**4117**	**PREDICTED: similar to Glyoxylase 1**	**REP**	**AB**	**AB**	**AB**	**AB**	**AB**	**AB**	**gi|50740506**	**20540.2**	**6.1**
**6402**	**creatine kinase M-type**	**AB**	**1.16**	**1.32**	2.64*	2.68**	2.47**	**−1.04**	**gi|45382875**	**43301**	**6.5**
**6408**	**creatine kinase M-type**	**−1.39**	2.07*	**1.75**	6.7**	**1.14**	**2.27**	**−1.06**	**gi|45382875**	**43301**	**6.5**
**6414**	**enolase**	**1.24**	**1.18**	**1.28**	**1.59**	2.38**	**1.4**	**1.25**	**gi|116248308**	**41047.2**	**5.62**
**9101**	**glutathione S-transferase**	**1.04**	**1.99**	**1.39**	2.47**	2.7**	2.58**	**−1.13**	**gi|49169816**	**25282.4**	**8.86**
**4210**	**PREDICTED: gemin 4**	**−1.58**	**1.18**	**1.56**	**−1.24**	**1.07**	−2.17**	**−1.37**	**gi|224076562**	**30154.9**	**4.96**
**1109**	**cathepsin B precursor**	**1.02**	**−1.03**	**1.04**	2.27**	2.39**	2.86**	**1.04**	**gi|46195455**	**37562.7**	**5.74**
**5607**	**peptidase D**	**−1.39**	**1.49**	**IN**	**1.73**	**IN**	2.08**	**−1.21**	**gi|169139269**	**55101.6**	**6.07**
**9111**	**peptidyl-prolyl cis-trans isomerase B precursor**	**1.15**	**1.55**	**1.12**	2**	**1.78**	**1.88**	**−1.17**	**gi|45382027**	**22398.8**	**9.4**
**6111**	**PREDICTED: similar to Glutathione S-transferase theta 1 isoform 3**	**1.55**	**−1.1**	**−1.59**	−2.38**	**−1.56**	−3.36**	**1.13**	**gi|118098704**	**27843.7**	**6.25**
**Immunity, apoptosis, tumor development and viral infection and replication**											
**6507**	**septin-6**	**−1.15**	**−1.27**	−6.03**	**−1.82**	**−1.35**	**−1.39**	**1.2**	**gi|71895629**	**48656.9**	**6.67**
**6521**	**septin-6**	**1.1**	**1.25**	2.42**	**−1.3**	**−1.05**	**−1.15**	**−1.55**	**gi|71895629**	**43021.1**	**6.63**
**7310**	**septin-9**	**−1.08**	**−1.38**	**−1.08**	−2.87**	−2.09*	−2.33*	**−1.25**	**gi|71897123**	**65482.8**	**8.42**
**3203**	**PREDICTED: similar to PTB-associated splicing factor**	**AB**	**AB**	−2.1**	**AB**	−2.75**	**−1.09**	2.06**	**gi|118101662**	**69173.9**	**9.43**
**7116**	**PREDICTED: heterogeneous nuclear ribonucleoprotein A3**	**−1.97**	2.16**	**−1.67**	11.96**	2.33**	2.4**	**1.4**	**gi|118093536**	**40603.2**	**9.24**
**8310**	**PREDICTED: heterogeneous nuclear ribonucleoprotein A3**	**AB**	**AB**	**AB**	−6.86**	**AB**	**AB**	**AB**	**gi|118093536**	**40603.2**	**9.24**
**1310**	**PREDICTED: heterogeneous nuclearribonucleoproteins C1/C2-like**	**−1.22**	**−1.19**	**−1.04**	−3.14**	−2.43*	**−1.36**	**1.29**	**gi|296224780**	**23551.2**	**9.87**
**8304**	**heterogeneous nuclear ribonucleoprotein A/B**	**1.02**	**1.29**	**−1.92**	−2.39**	−2.58**	−2.11**	**−1.18**	**gi|45384514**	**31841.8**	**8.62**
**2613**	**heterogeneous nuclear ribonucleoprotein H**	**−1.14**	**−1.78**	**1.87**	−2.13**	**−3.36**	**−2.48**	**1.05**	**gi|45383173**	**56530.6**	**5.38**
**2610**	**heterogeneous nuclear ribonucleoprotein H**	**1.22**	−2.25*	**−1.31**	**AB**	−2.11**	**−1.82**	**−2.91**	**gi|45383173**	**56530.6**	**5.38**
**3101**	**heterogeneous nuclear ribonucleoproteins A2/B1**	**1.69**	**1.46**	**−1.39**	**−1.6**	−2.00**	**1.79**	**1.07**	**gi|71896753**	**36962.4**	**8.67**
**8211**	**heterogeneous nuclear ribonucleoproteins A2/B1**	**−1.1**	**−1.11**	**1.06**	−2**	−2**	**−1.68**	**−1.12**	**gi|71896753**	**36962.4**	**8.67**
**8213**	**heterogeneous nuclear ribonucleoproteins A2/B1**	**1.04**	**−1.07**	**−1.46**	−2.53**	**−1.41**	−2.01**	**−1.1**	**gi|71896753**	**36962.4**	**8.67**
**9205**	**heterogeneous nuclear ribonucleoproteins A2/B1**	**−1.08**	**1.07**	**−1.41**	−2.47**	−2.19**	**−1.57**	**−1.12**	**gi|71896753**	**36962.4**	**8.67**
**7006**	**macrophage migration inhibitory factor**	**1.35**	**−1.03**	−2.04**	**−1.64**	**−1.77**	**−1.84**	**1.74**	**gi|212258**	**12704.2**	**6.88**
**2905**	**heat shock protein HSP 90-alpha**	**−1.37**	**−1.55**	**−1.23**	−3.73**	**−1.58**	**−1.63**	**−1.67**	**gi|157954047**	**84006.5**	**5.01**
**7215**	**annexin A1**	**AB**	**IN**	**1**	**IN**	**IN**	**IN**	**AB**	**gi|46195459**	**38475.9**	**7.05**
**7703**	**far upstream element-binding protein 1**	**1.03**	**−2.56**	**1.34**	−2.53**	−2.38*	**−1.85**	**−1.48**	**gi|83320094**	**67155.3**	**7.18**
**3014**	**stathmin**	**1.19**	**1**	**−1.16**	−4.27**	−7.72**	−7.18**	**1.05**	**gi|50053682**	**17071.9**	**6.18**
**4003**	**stathmin**	**1.1**	−2.1**	**−1.1**	−4.91**	−4.49**	−5.08**	**−1.1**	**gi|50053682**	**17071.9**	**6.18**
**405**	**vimentin**	**−1.5**	3.27*	**1.56**	3.31**	**1.36**	**1.06**	**1.04**	**gi|114326309**	**53109.6**	**5.09**
**1605**	**vimentin**	**1.62**	**1.76**	**1.77**	2.9**	**−1.35**	**−1.08**	**−1.01**	**gi|114326309**	**53109.6**	**5.09**
**409**	**vimentin**	**1.23**	**1.64**	**1.42**	2.26**	**1.47**	**1.31**	**1**	**gi|114326309**	**53109.6**	**5.09**
**205**	**nucleolar protein B23/No38**	**1.28**	**1.76**	**−1.51**	**1.54**	**1.58**	**1.65**	6.95**	**gi|212456**	**10716.2**	**4.38**
**6003**	**beta-galactoside-binding lectin**	**1.07**	2.22**	**1.56**	2.7**	2.66**	2.4**	**−1.45**	**gi|45382785**	**15053.5**	**6.58**
**3113**	**PREDICTED: similar to natural killer cell enhancing factor isoform 4**	**1.63**	**1.12**	1.63*	**1.58**	2.5**	2.28**	**−1.27**	**gi|50751518**	**22300.5**	**8.24**
**5304**	**unnamed protein product**	**1.23**	**1.88**	**1.45**	**−1.49**	**−1.26**	**1.28**	2.11**	**gi|74142813**	**50433.1**	**6.17**
**1104**	**PREDICTED: similar to interferon, gamma-inducible protein 30**	**1.14**	**−1.05**	**−1.04**	−2.27**	**−1.29**	**−1.12**	**1.19**	**gi|50761132**	**14269.9**	**5.73**
**401**	**mCG49244**	**1.23**	**−1.56**	**1.27**	−2.25*	−2.04**	**−1.63**	**−1.73**	**gi|148694498**	**21747.1**	**5.71**
**114**	**SET**	**1.62**	5.16**	**1.18**	**−1.01**	**−1.07**	**1.01**	**1.51**	**gi|3953617**	**24348**	**4.97**
**Mitosis, replication and translation**											
**1402**	**put. beta-actin (aa 27–375)**	**1.01**	**−1.27**	−2.74**	−13.22**	−14.21**	**−1.1**	**−1.07**	**gi|49868**	**39160.6**	**5.78**
**2303**	**suppressor of G2 allele of SKP1 homolog**	**AB**	−2.33*	**−1.1**	**REP**	**REP**	**REP**	**REP**	**gi|71895155**	**20497.5**	**6.35**
**6307**	**mitotic checkpoint protein BUB3**	**AB**	−3.53**	**1.61**	**REP**	**REP**	**REP**	−2.97**	**gi|57529813**	**37256.3**	**6.5**
**3501**	**PREDICTED: similar to eukaryotic translation initiation factor 4H isoform 1**	**1.26**	−2.31*	**−1.33**	**−1.99**	**−1.45**	**−1.84**	**1.02**	**gi|126314438**	**27406.4**	**6.78**
**8202**	**PREDICTED: similar to eukaryotic translation initiation factor 4H isoform 1**	**1.17**	−3.81**	**1.13**	−2.86**	**−1.72**	**−1.38**	−2.65**	**gi|126314438**	**27406.4**	**6.78**
**2013**	**PREDICTED: similar to histone H2B**	3.97*	**REP**	**REP**	**REP**	**REP**	**REP**	8.73**	**gi|149617840**	**29704.7**	**9.68**
**7117**	**single-strand binding protein**	**AB**	**AB**	**AB**	**IN**	**IN**	**IN**	**AB**	**gi|42523087**	**15766.8**	**6.13**
**5603**	**pre-mRNA-processing factor 19**	**1.61**	**−1.34**	−2.62**	**−1.23**	**−1.09**	**−1.32**	**−1.16**	**gi|86129600**	**55100.2**	**6.19**
**Signal transduction**											
**3616**	**Amb7**	**1.22**	**1.9**	**1.94**	4.4**	**1.87**	**IN**	**AB**	**gi|117168610**	**225516.8**	**5.61**
**5518**	**coronin-1C**	**AB**	**AB**	**AB**	**IN**	**AB**	**AB**	**AB**	**gi|86129440**	**53174.1**	**6.22**
**6102**	**coronin-1C**	**−1.1**	**1.32**	**−1.33**	2.41**	3.78*	3.37**	3.76**	**gi|86129440**	**53174.1**	**6.22**
**3015**	**fatty acid-binding protein, adipocyte**	−2.05**	2.38*	2.34**	4.91**	4.73**	2.39**	**1.11**	**gi|45383556**	**14884.6**	**6.34**
**3012**	**fatty acid-binding protein, heart**	**−1.23**	**−1.49**	**AB**	9.97**	**1.5**	**1.62**	**1.77**	**gi|71894843**	**14806.6**	**5.92**
**3709**	**Lipocalin precursor**	**1.75**	**−1.47**	**1.09**	4.11**	6.62**	3.26*	**1.47**	**gi|225716896**	**20257.1**	**7.6**
**6213**	**PREDICTED: similar to GADS protein**	**−1.46**	**−1.24**	**1.56**	−2.49**	**1.7**	**−1.08**	**1.19**	**gi|118082689**	**33178.5**	**6.23**
**5205**	**PREDICTED: similar to GADS protein**	**−1.11**	−2.14**	**−1.15**	−3.84**	**1.04**	**−1.16**	**−1.45**	**gi|118082689**	**33178.5**	**6.23**
**6310**	**PREDICTED: similar to nucleic acid binding protein isoform 2**	**1.42**	**−1.6**	**−1.03**	**−1.41**	−2.07**	**−1.59**	**−1.63**	**gi|126345445**	**35784.3**	**7**
**6011**	**PREDICTED: similar to retinoid binding protein 7**	−4.75**	**IN**	**IN**	**IN**	**IN**	4.34**	**AB**	**gi|118101075**	**14701.2**	**5.31**
**1008**	**thioredoxin**	**−1.26**	**−1.11**	**−1.02**	2.46*	**1.28**	**1.7**	**1.07**	**gi|45382053**	**11692.8**	**5.1**
**Transport**											
**6517**	**ferritin heavy chain**	**−1.14**	2.81**	**−1.44**	2.13**	4.11**	2.25**	**1.02**	**gi|45384172**	**21078.3**	**5.78**
**7203**	**ferritin heavy chain**	−4.57**	**1.42**	**−1.18**	3.89**	2.12**	2.49**	**1.03**	**gi|45384172**	**21078.3**	**5.78**
**8018**	**hemoglobin alpha-A chain**	−3.13**	**1.13**	**1.41**	**1.37**	**1.2**	**1.4**	**1.26**	**gi|4894665**	**10914.7**	**7.19**
**8003**	**hemoglobin alpha-A chain**	−2.54**	**−1.15**	**−1.12**	**−1.03**	**1.28**	**−1.14**	**1.06**	**gi|4894665**	**10914.7**	**7.19**
**6009**	**hemoglobin subunit alpha-D**	**−1.08**	**−1.07**	3.59**	**1.2**	**1.02**	**1.04**	**1.03**	**gi|52138645**	**15685**	**7.01**
**6804**	**ovotransferrin BB type**	**1.03**	**1.32**	**−1.27**	2.71*	**1.82**	**1.78**	**1.03**	**gi|71274075**	**77781.4**	**6.85**
**6810**	**ovotransferrin BB type**	**AB**	**1.95**	**REP**	20.2*	**IN**	**IN**	**AB**	**gi|71274075**	**77781.4**	**6.85**
**6801**	**ovotransferrin CC type**	**1.56**	**1.27**	**1.19**	2.52**	2.89*	2.48*	**−1.17**	**gi|71274077**	**77750.4**	**7.08**
**7201**	**mediator of cell motility 1**	**1.37**	**−1.06**	**1.02**	−2.93**	**−1.09**	**1.54**	**−1.24**	**gi|300676818**	**31517.5**	**6.49**
**Others proteins**											
**6104**	**603815993F1 CSEQCHN52 Gallus gallus cDNA clone ChEST809j23 5′, mRNA sequence**	**1.14**	**−1.17**	**−1.57**	−2.53**	−2.12**	**−1.94**	**1.19**	**gi|25541234**	**23338.9**	**6.03**
**304**	**alpha-tropomyosin (partial)**	**−1.32**	**1.18**	**1.12**	2.37**	2.38**	2.46**	**1.71**	**gi|212815**	**30045.3**	**4.64**
**5815**	**conalbumin**	**−1.17**	**1.11**	**−1.05**	5.97*	3.44**	**1.9**	**−1.12**	**gi|295721**	**79551.1**	**6.85**
**4012**	**cytoChrome c subfamily, putative**	**AB**	**IN**	**AB**	**IN**	**IN**	**26.4**	**AB**	**gi|124005227**	**24128.2**	**9.21**
**307**	**putative nucleophosmin 1 variant 1**	**1.2**	−2.66*	**1.23**	−2.09**	−3.13**	**−1.19**	**1.06**	**gi|45383996**	**32612**	**4.66**
**4412**	**putative protein product of Nbla10058**	**AB**	**IN**	**AB**	**AB**	**AB**	**AB**	**AB**	**gi|76879893**	**48587.2**	**5.85**
**204**	**PREDICTED: similar to Acidic leucine-rich nuclear**	**1.3**	**−1.71**	**−1.6**	−2.22*	−3.2**	−2.45**	**−1.14**	**gi|118096008**	**32075.9**	**3.98**
**4420**	**PREDICTED: similar to cyclophilin**	**AB**	**AB**	**AB**	**IN**	**IN**	**AB**	**AB**	**gi|118089782**	**39402.9**	**5.61**
**6304**	**PREDICTED: similar to KIAA0089**	**1.01**	**−1.19**	**−1.45**	**−1.43**	−2.6**	**−1.8**	**1.2**	**gi|50732786**	**38564.1**	**6.55**
**4116**	**PREDICTED: similar to LOC129607 protein**	**−1.25**	**IN**	**AB**	**IN**	**IN**	**IN**	**AB**	**gi|118088982**	**31865.5**	**8.45**
**4114**	**PREDICTED: similar to MGC84496 protein**	**−1.43**	**−1.32**	**−1.37**	**1.17**	**1.64**	**1.98**	2.77**	**gi|118101652**	**22693.7**	**6.07**
**8002**	**protein S100-A10**	**−1.27**	**1.37**	**1.11**	3.15*	**1.27**	**1.3**	**1**	**gi|45382861**	**11282.5**	**6.82**
**1006**	**transthyretin precursor**	**−1.12**	**−1.01**	**1.35**	−2.64**	−3.31**	**−1.57**	**−1.09**	**gi|45384444**	**16299.1**	**5.11**
**Hypothetical proteins**											
**3507**	**PREDICTED: hypothetical protein**	**1.52**	**−1.52**	**−1.24**	−3.08**	−2.04*	**−1.49**	**−1.31**	**gi|50762370**	**47124.5**	**5.64**
**2203**	**PREDICTED: hypothetical protein**	**−1.37**	**−1.72**	**−1.72**	−6.61**	−2.27**	−3.83**	**−1.02**	**gi|118087111**	**30122.5**	**5.51**
**2209**	**PREDICTED: hypothetical protein**	**−1.12**	**−1.22**	−2.19**	**−1.3**	**−1.27**	**1.14**	**1.43**	**gi|118092623**	**32616.1**	**5.53**
**3110**	**PREDICTED: hypothetical protein**	**AB**	−2.73**	**IN**	**AB**	−3.04**	−2*	**−1.22**	**gi|118084734**	**18881.6**	**6.08**
**5108**	**PREDICTED: hypothetical protein**	−5.55**	**REP**	**REP**	**−1.2**	**IN**	**REP**	**1.75**	**gi|118084734**	**18881.6**	**6.08**
**7214**	**PREDICTED: hypothetical protein**	**−1.48**	**1.03**	−2.13**	**−1.46**	**1.88**	**1.06**	**−1.04**	**gi|50762370**	**47124.5**	**5.64**
**2307**	**hypothetical protein**	**−1.24**	**1.82**	**1.47**	**1.39**	**1.55**	2.28*	2.19**	**gi|53126859**	**42107.9**	**5.31**
**4013**	**hypothetical protein Bsuib36_09931**	**AB**	**AB**	**AB**	**IN**	**AB**	**AB**	**AB**	**gi|254704229**	**122393.8**	**8.07**
**3210**	**hypothetical protein PANDA_003704**	**AB**	**AB**	**AB**	**IN**	**AB**	**AB**	**AB**	**gi|281343027**	**31307.8**	**5.44**

Fold change = infected/control. Positive indicates up regulation, negative indicates down-regulation.

IN = induced, detected only in infected group, REP = repressed, detected only in the control group, AB = absent in both groups, dpi = days post-infection.

Asterisks (*) indicates statistically significant difference. (*) indicates p<0.05 and (**) indicates p<0.01.

Based on the GO annotations, more than 50% of the associated biological processes were metabolic processes (GO:0008152, 23.4%), regulations of biological processes (GO:0050789, 17.3%) and responses to stimuli (GO:0050896, 12.7%). In addition, the majority of the associations, with respect to molecular function, were with GO terms such as protein binding (GO:0005515, 27.2%), nucleic acid binding (GO:0003676, 13.6%) and hydrolase activity (GO:0016787, 12.3%). Meanwhile, 7.0% of the proteins were associated with functions such as signal transduction, antioxidation, transcription regulation and receptor binding.

### Analysis of the protein functional interaction networks

**Figure 4:**
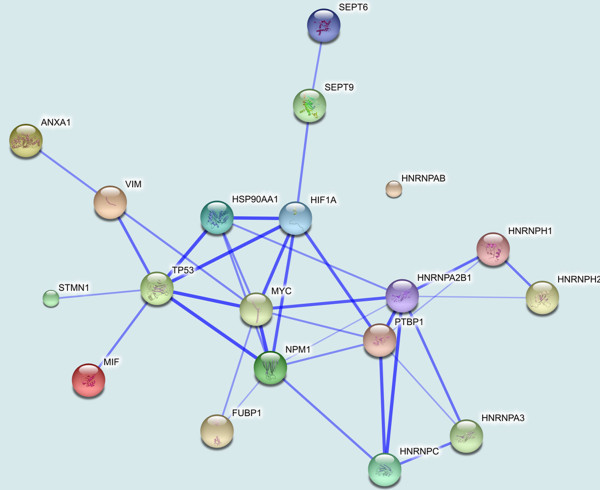
**Based on the STRING database, the network of protein–protein interactions in the MDV-infected thymuses.** Sixteen differentially expressed proteins formed a network map with the tumour protein p53 (TP53), myc proto-oncogene protein (MYC) and hypoxia-inducible factor 1-alpha (HIF1A) at its core.

The STRING Database was used to establish possible interactions among the detected proteins. In this analysis, 16 tumour proteins were identified by interaction analysis, and a network map with the tumour protein p53 (TP53), myc proto-oncogene protein (MYC) and hypoxia-inducible factor 1-alpha (HIF1A) at its core was obtained (Figure 4).

### Validation of the mRNA expression levels using real-time PCR

**Figure 5:**
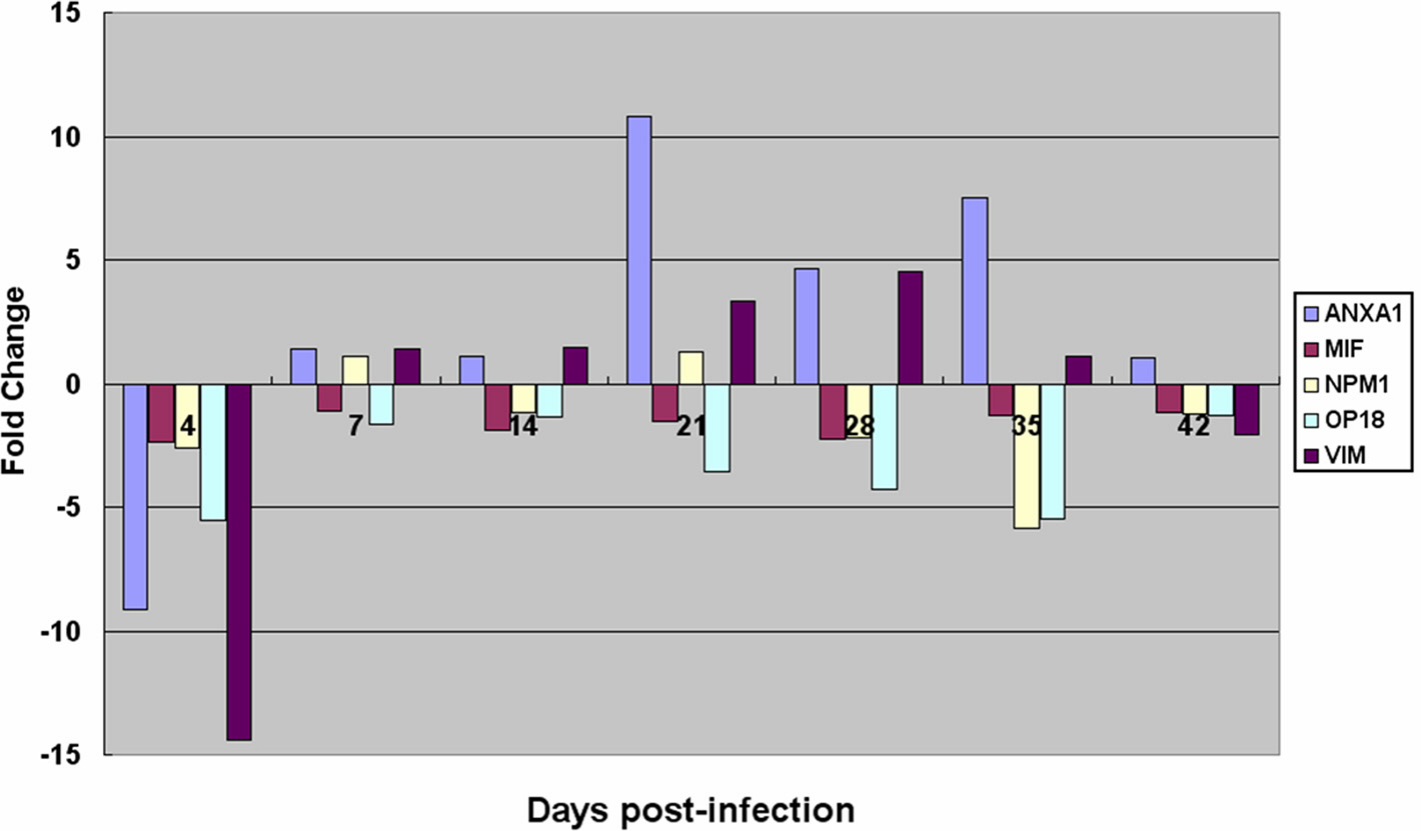
**mRNA changes for the five proteins that were significantly altered in response to the MDV infection at the different stages.** The y-axis is the fold change, with the positive values representing up-regulation and the negative values representing down-regulation. The abscissa axes represent different stages.

To validate the results of the proteomic analysis, the expression levels of ANXA1, MIF, NPM1, OP18 and VIM in the chicken thymus were determined using real-time PCR. The mRNA expression levels of these proteins were down-regulated at 4 dpi and then remained down-regulated for MIF and OP18 and up-regulated for ANXA1 and VIM (Figure 5). These results are consistent with the results of our proteomic analysis (see Table 1 and Figure 5).

### Viral infection levels in the thymuses of the MDV-infected chickens

**Figure 6:**
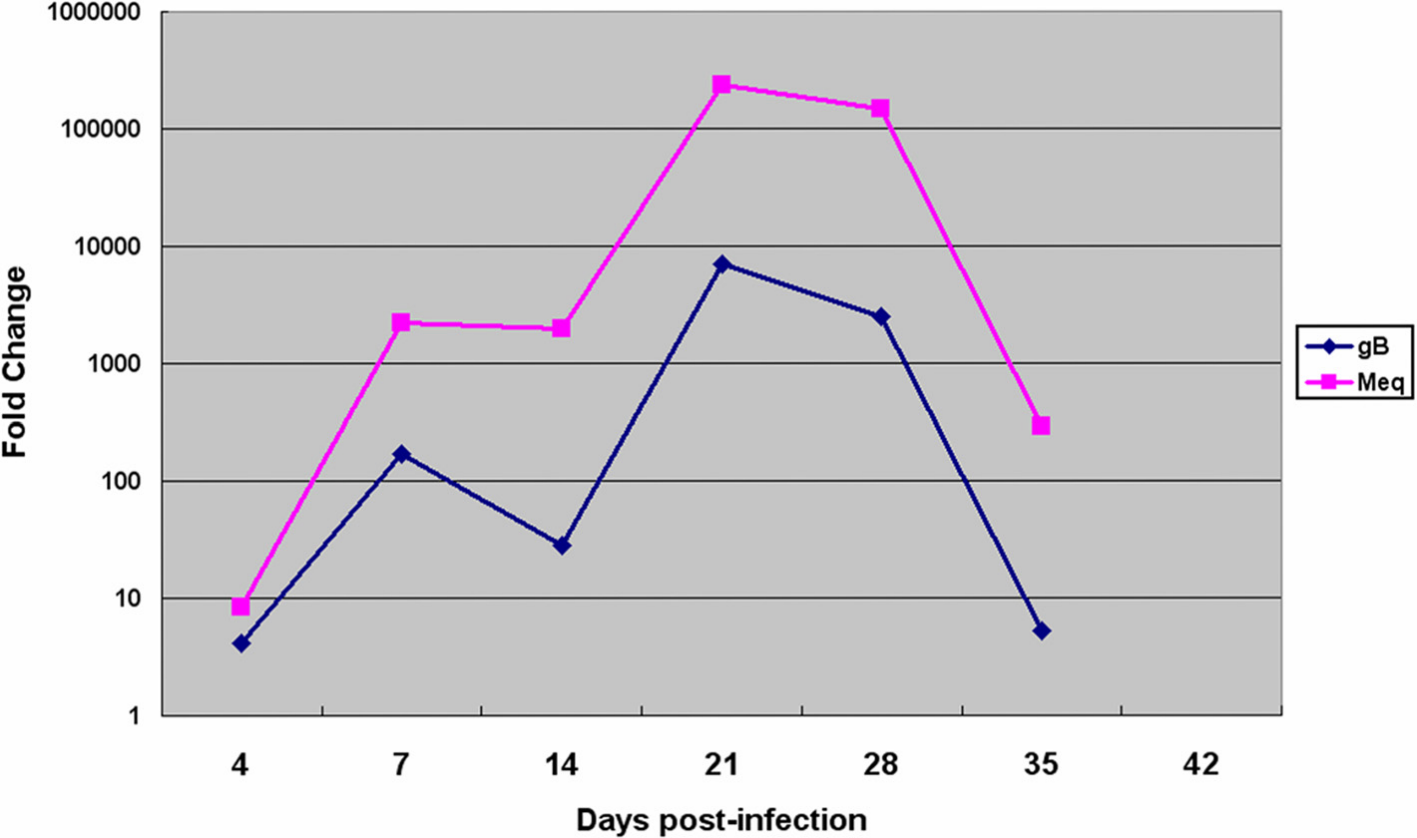
**mRNA levels of the MDV transcripts in the chicken thymuses infected with RB1B.** The expression levels of Meq and gB mRNA were detected using real-time PCR and calculated using the 2 –^ΔΔ^CT method.

The MDV infection levels in the thymus at seven points were detected using real-time PCR and are shown in Figure 6. The expression level of gB was transiently increased at 4 and 7 dpi and decreased at 14 dpi, the latent infection phase. The gB expression level was increased again at 21 dpi, suggesting the occurrence of MDV reactivation and transformation, and decreased at 28 dpi. These changes were consistent with the numbers of differentially expressed proteins. After 28 dpi, the mRNA level of gB in the MDV-infected chickens displayed a rapid decrease, and little mRNA was detected at 42 dpi. These changes coincided with the Meq expression levels.

## Discussion

The thymus is the specific organ in which the maturation and differentiation of avian T-lymphocytes take place. T lymphocytes, or T cells, are of a key importance to the immune system and are at the core of adaptive immunity. Chickens infected with MDV display thymic atrophy (Figure 1A) and eventually the formation of T-cell lymphoma. However, little is known about the underlying mechanisms of this phenomenon. In the present study, 119 proteins differentially expressed in thymus specimens from chickens infected with MDV were identified by two-dimensional electrophoresis and mass spectrometry at seven time points. The chicken thymuses displayed severe atrophy at 21, 28 and 35 days after infection with RB1B, and their size gradually returned to normal at 42 dpi. The change in thymus size during the course of the MDV infection may have influenced the expression of cellular structural proteins, enzymes and cytoskeleton proteins. However, we should also note that the 2-DE and real-time PCR analyses were conducted using the same amounts of protein and mRNA, which might exclude the effect of thymus size to some extent. In addition, the change in the cellular composition of the thymus could be affected the total proteome. The thymic atrophy in the chickens infected with MDV has a significant influence on host immune suppression. Permanent immunosuppression tends to correlate with the eventual development of tumours, which enhances our understanding of the mechanisms of T-lymphoma formation.

To better understand host responses to MDV infection, the expression of viral genes (gB and Meq) was detected using real-time PCR (Figure 6). We found a good correlation between viral gene product and host differentially expressed proteins. The expression levels of gB and Meq were transiently increased at 4 and 7 dpi and decreased at 14 dpi during the latent infection period. The gB and Meq expression levels were again increased at 21 dpi, suggesting the occurrence of MDV reactivation and transformation (Figure 6). At 21 dpi, the over-expressed viral gene products led to serious changes in protein expression levels and thymic atrophy (Figures 1, 3 and 6). These changes could be due to the disease pathology induced by MDV infection. Consistent with the MDV pathogenesis, early cytolytic infection occurred at 3 to 7 dpi and then entered latency, followed by the proliferative/transformation phase, leading to lymphoma formation at approximately 21 to 28 dpi. After 28 dpi, the mRNA levels of gB and Meq in the MDV-infected chickens displayed a rapid decrease, and little mRNA was detected at 42 dpi. This change may be due to the cellular response of lesion regression. It has been reported that lymphomas can occur at any time from approximately 3~4 weeks, and lesion regression can occur after lymphomas [13].

More interestingly, more than 20 differentially expressed proteins were directly associated with immunity, apoptosis, tumour development and viral infection and replication (Table 1). Notably, at least nine proteins were identified for the first time in this study: macrophage migration inhibitory factor, heat shock protein 90-alpha, annexin A1, far upstream element-binding protein 1, septin-6, septin-9, beta-galactoside-binding lectin, mCG49244 and an unnamed protein product. Among these proteins, some formed a network map encompassing TP53, MYC and HIF1A at its core and are directly associated with immunity, apoptosis, tumour development and viral infection and replication (Figure 4). Within this map, we found that MIF, HSP90AA1, NPM1, STMN1(OP18) and VIM can interact with the tumour suppressor protein TP53. It has been reported that the Meq oncoprotein directly interacts with p53 and inhibits p53-mediated transcriptional activity and apoptosis [14]. This scenario could provide an interesting link between MEQ and these proteins and enhance our understanding of MDV pathogenesis. The mRNA levels of these proteins were confirmed by real-time PCR (Figure 5), and some of these proteins are further discussed in this study.

### Roles of the differentially expressed proteins in MDV infection

After MDV infection, the expression of the MIF protein displayed a slight increase at 4 dpi, followed by a down-regulation and subsequent increase at 42 dpi. The mRNA expression level of MIF was down-regulated at all stages, which may have resulted for two reasons. First, different mechanisms control the transcription and translation of the MIF gene in chicken thymus, and the mRNA abundance is not always consistent with the protein level. Second, it is possible that the transcription level of the MIF gene is easily influenced by MDV during the early stages of the infection. However, MIF is a pro-inflammatory factor, and its translational level is enhanced by the host’s regulatory mechanisms. Notably, the differences in the expression levels of the MIF mRNA and protein could be involved in the MDV infection. It is believed that macrophages transfer the virus to B cells, which are the primary target cells that are infected between 3 and 6 days post-infection [4]. A heavy infiltration of lymphocytes and macrophages occurs around blood vessels at 8–10 days post-MDV infection [15], and a study has demonstrated that some new MDV strains can replicate in macrophages, which leads to increased macrophage death [16]. MIF is involved in virus infection, and its target cells are mainly macrophages. It has been reported that human cytomegalovirus (HCMV), a herpesvirus, paralyses macrophage motility through the down-regulation of chemokine receptors, reorganisation of the cytoskeleton, and release of MIF [17]. MIF also promotes HIV-1 replication through the activation of HIV-1 long terminal repeats (LTR) [18]. Increased MIF at 4 dpi may promote MDV replication. The down-regulation of MIF indicates a strong macrophage migration activity, and macrophages that carry MDV could spread the MDV infection to other cells. Another protein, Anx-A1, was induced at 7, 21, 28 and 35 dpi and may be associated with MDV infection. Research studies suggest that the annexin family members are involved in the viral replication cycle, which may integrate the regulation of virus infection by forming networks [19]. Nucleolar protein B23 (NPM1), an acidic nucleolar protein, was up-regulated in the thymus of chickens infected with MDV and may indicate the nuclear importation of MDV. It has been demonstrated that NPM1 stimulates the nuclear importation of the HIV-1 Rev protein [20], and NPM1 is also an important factor for the nucleolar localisation of the HIV protein Tat [21]. The reduction in the Hsp90alpha level during early MDV infection might be related to a latent infection. Hsp90 plays an important role in the replication and infectivity of some herpesviruses, such as herpes simplex virus type 1 [22], Epstein–Barr virus (EBV) [23] and Kaposi’s sarcoma-associated herpesvirus (KSHV) [24], and is required for the folding, stability and intracellular transport of multiple viral proteins and for the activity of viral polymerases. Taken together, our observations suggest that changes in the levels of these proteins influence MDV replication and infection. However, the specific mechanisms are unknown and will require further study.

### The chicken immune response to MDV infection

Suppression of the immune response by MDV infection is a critical feature of the disease. MIF down-regulation may contribute to the inhibition of the immune response. MIF, a classic pro-inflammatory cytokine and a pivotal regulator of innate immunity, promotes innate and adaptive immune responses through the activation of macrophages and T cells [25,26]. Moreover, it directly inhibits the immunosuppressive actions of glucocorticoids [27] through the suppression of mitogen-activated protein kinase (MAPK) phosphatase-1 (MKP-1) [28-30]. MKP-1, which is induced by glucocorticoids, inactivates the proinflammatory ERK1/2, JNK, and p38 pathways. Anx-A1, a pivotal regulator of the innate and adaptive immune systems, also promotes immunosuppression [31]. This protein was induced at 7, 21, 28 and 35 dpi and has a powerful suppressive effect on the innate immune system. Recent investigations on the role of this protein in the adaptive immune response have revealed a previously unknown ‘dark side’ to this protein, that is, it is a positive modulator of T cell activation [32]. High levels of Anx-A1 influence the differentiation of T cells *in vivo*, and hence, may contribute to the development of T-cell-driven autoimmune diseases.

While heat shock proteins are molecular chaperones, they have also been implicated in the stimulation of the innate and adaptive immune systems [33,34]. Recent biochemical evidence highlights the role of Hsp90alpha in the endogenous processing of MHC class I antigens, and the absence of Hsp90alpha results in the decreased surface expression of MHC I [35]. Levy et al. [36] reported that RB1B was able to markedly decrease MHC class I expression. In addition, those authors found that MDV pUL49.5 directly down-regulates MHC class I expression [37], and beta2 microglobulin was also decreased in the bursa of Fabricius of chickens infected with RB1B [6]. A subsequent gene expression study indicated a down-regulation of MHC class II expression in the spleens of MDV-infected chickens [9]. The specific relationship between the down-regulation of HSP90A and MHC class I expression during the course of MDV infection is not yet clear. However, these findings indicate that a potential mechanism of immune evasion mediated by MHC expression on cell surfaces might be employed by MDV. In addition, the immune suppression that occurs after MDV infection is not only related to changes in immune-related proteins but may also be associated with immune evasion.

### Apoptosis and thymic atrophy after MDV infection

The thymic atrophy of chickens infected with MDV is the most significant manifestation of immune suppression and might be related to apoptosis. Morimura et al. reported that MDV can induce apoptosis and the down-regulation of CD8 molecules on peripheral CD4+ T cells and in the thymus, which could contribute to immune suppression [10-12]. In this study, although we did not detect cell apoptosis by flow cytometry, changes in some proteins indicated apoptosis in the thymuses of the chickens. According to the network of protein–protein interactions in the thymuses infected with MDV, five differentially expressed proteins can interact with P53 (Figure 6). The decreased levels of stathmin/oncoprotein18 (Op18) and MIF may increase the level of p53 and promote p53-mediated apoptosis before T-cell lymphoma formation. It has been demonstrated that MIF suppresses the expression of p53 and its activity [26,38]. MIF increases resistance to apoptosis by activating the nuclear factor-kappa B (NF-κB) system and repressing the function of p53 [39]. Changes in Op18 may be related to the increase in P53 because p53 is associated with the negative regulation of stathmin expression [40-42]. In the thymus, this protein was mainly down-regulated and was significantly differentially expressed at 21, 28 and 35 dpi. Lu et al. [6] also reported that Op18 was significantly reduced at 4, 7 and 21 dpi in the bursa of Fabricius of chickens infected with RB1B. In addition, ANX-A1 is relevant to the regulation of cell growth and apoptosis [43,44], and ANXA1 overexpression has been shown to promote apoptosis [45,46]. It has also been reported that ANXA1 expression in leukaemic cells mediates the engulfment of apoptotic cells by macrophages [47]. In short, MDV infection results in immune suppression and the induction of apoptosis, which eventually leads to thymic atrophy.

### T-cell lymphoma formation during MDV infection

The Meq oncoprotein of the Marek’s disease virus is the major oncogene involved in the induction of tumours and inhibits p53 transcriptional and apoptotic activities by interacting with p53 [14]. Permanent immunosuppression tends to correlate with the eventual development of tumours. The role of host proteins associated with tumour growth and metastasis in the formation of T-cell lymphoma cannot be ignored. MIF, a negative regulator of the important tumour suppressor p53, is involved in tumour occurrence and evolution [48-50]. A significant amount of evidence indicates that MIF influences several important biological mechanisms and processes by which tumours thrive and spread. One of the most important of these mechanisms is the modulation of hypoxic adaptation within the tumour microenvironment through the direct promotion of the hypoxia-induced stabilisation of HIF-1α [51]. We speculate that the increased level of MIF protein observed at 42 dpi may indicate hypoxic adaptation within the tumour microenvironment. Anx-A1 is directly related to tumour development [52]. This protein was induced at 7, 21, 28 and 35 dpi and could be a key host factor that enhances the formation of T-cell lymphomas. Strong evidence for this process is provided by the finding that the increased expression of Anx-A1 promotes tumour growth, invasion and metastasis in gastric carcinoma [53], melanoma [54], breast cancer [55] and colorectal cancer [56]. Using AnxA1- knock out (KO) mice, it has been determined that tumour growth and metastasis are significantly decreased, whereas rodent survival and tumour necrosis are significantly increased when tumours grow in AnxA1-KO mice [57]. In addition, the up-regulation of several cytoskeletal network proteins, e.g., vimentin, beta-actin and keratin (type I cytoskeletal 15), also promotes tumour growth and metastasis. Research suggests that vimentin, a major intermediate filament (IF) protein of mesenchymal cells, is very important for tumour growth and metastasis [58,59]. Beta-actin specifically controls cell growth and migration [60], and an increase in beta-actin levels correlates with a higher level of invasiveness for a select hepatoma in Morris 5123 cells [61]. Changes in these proteins indicate that MDV infection and T-cell lymphoma formation involve the host cytoskeleton. Nucleolar protein B23 (NPM1) plays multiple roles in cell growth and proliferation [62]. Thanthrige-Don et al. also reported an up-regulation of beta-actin and NPM1 in the spleens of MDV-infected chickens [5]. Interestingly, a report has shown that the interaction between Meq and Hsp70 is significant during MDV oncogenesis [63]. However, the biological consequences of the Meq–Hsp70 interaction are not clear. Finally, HSP90A was reduced at 21 dpi; however, whether HSP90 can interact with Meq and affect Meq carcinogenicity remains to be studied.

In addition, septin 9 and septin 6 levels were significantly decreased in the thymuses of chickens infected with MDV. Septins are a highly conserved family of GTP-binding cytoskeletal proteins implicated in oncogenesis [64,65]. Septin 9 (SEPT9), a DNA methylation-based biomarker, has been functionally linked with oncogenesis through its activation of the hypoxia-inducible factor-1 (HIF-1) pathway, which promotes tumour progression, and the c-Jun-N-terminal kinase (JNK) pathway, which plays an important role in cell proliferation, cell transformation, and tumour progression [66,67]. The differential expression of heterogeneous nuclear ribonucleoproteins (hnRNPs), including HnRNP A / B, HnRNP A2/B1, HnRNP H, and HnRNP C1/C2, was also detected in this study. These proteins play key roles in tumour development and progression [68]. Studies have shown that hnRNP A2 / B1 and hnRNP A1 can combine their telomere DNA sequences and that their encoded protein isoforms can interact with the telomerase. Tumour cells fail to enter senescence due to their telomere lengths, which are maintained by telomere-bound proteins that recruit the enzyme telomerase [68,69]. Currently, the roles of septins and hnRNPs in MD tumour progression are not clear and require further study.

## Conclusions

This paper provides a proteomic profiling of host responses to MDV in the thymus of chickens. Changes in protein levels partially elicited the mechanisms involved in MDV oncogenesis and pathogenesis. The functions of these proteins will be verified in our future works.

## Methods

### Experimental animals and virus

All chickens used in this study were 1-day-old specific-pathogen-free white Leghorns obtained from Merial Vital (Laboratory Animal Technology Co., Ltd., Beijing, China). The chickens were housed in an isolation facility at the College of Veterinary Medicine, Yangzhou University. The RB1B strain of very virulent MDV was maintained in the laboratory.

### Experimental design

The experimental work was performed as reported previously [6]. Briefly, a total of 48, 1-day-old birds were randomly divided into infected and uninfected control groups and kept in separate units under similar environmental conditions. The infected group (n = 24) was given 800 plaque-forming units of the RB1B virus intraperitoneally at 1 day old. At 4, 7, 14, 21, 28, 35 and 42 dpi, six chickens (three infected and three uninfected control birds) were sacrificed, and the whole thymus was excised rapidly, rinsed with ice cold phosphate-buffered saline (PBS, pH 7.4) to remove blood contaminants and immediately stored in liquid nitrogen until the proteomic and real-time PCR analyses. The animal experiments were conducted in accordance with the guidelines provided by the Chinese Council on Animal Care. All experiments complied with institutional animal care guidelines and were approved by the University of Yangzhou Animal Care Committee (protocol number 06R015).

### Sample preparation

The samples of thymus protein were prepared as previously described [6]. Frozen thymus tissue was ground into a fine powder in liquid nitrogen with a pre-chilled mortar and pestle. This ground tissue was used for both the protein and real-time PCR analyses. Five milligrams of ground tissue was dissolved directly in 1.0 ml of extraction buffer (8 M urea, 2 M thiourea, 2% CHAPS, 60 mM DTT (dithiothreitol), 0.2% Bio-Lyte 3∕10 ampholyte, 0.1% Bio-Lyte 5∕8 ampholyte and 0.001% bromophenol blue) and shaken on ice for 2 h. A cocktail of protease inhibitors (Sigma) was added every two hours during sample preparation to protect the proteins from degradation. The homogenate was sonicated, gently shaken on ice for 4 h and subsequently centrifuged at 16,000 *g* (5415 R Eppendorf) for 70 min (16°C). The supernatant was collected and stored at −20°C. Protein concentrations were determined using the Bradford method [70].

### 2-DE

Protein sample (300 μg) was incubated for 30 min at 20°C in 400 μl rehydration buffer (8 M urea, 2 M thiourea, 2% CHAPS, 60 mM DTT, 0.2% Bio-Lyte 5∕8 ampholyte, 0.2% Bio-Lyte 3∕10 ampholyte and 0.001% bromophenol blue) and centrifuged at 16,000 *g* (5804 R Eppendorf) for 10 min (16°C). The supernatant was collected, and ReadyStrip IPG strips (17 cm, pH 3–10 nonlinear gradient, Bio-Rad) were rehydrated overnight at 20°C in a PROTEAN IEF focusing tray (Bio-Rad, USA). After rehydration, isoelectric focusing (IEF) was performed at 20°C using a Protean IEF Cell (Bio-Rad, USA) and the following conditions: 250 V, slow, 0.5 h; 500 V, linear, 0.5 h; 1000 V, rapid, 1 h; 8000 V, linear, 4 h; 8,000 V, rapid, 55,000 V-hr; 500 V, rapid, any time. After IEF, the IPG strips were incubated in SDS-PAGE equilibration buffer I (6 M urea, 0.375 M Tris–HCl, pH 8.8, 2% (w/v) SDS, 20% (v/v) glycerol and 2% (w/v) DTT) and buffer II (the DTT was replaced with 2.5% (w/v) iodoacetamide), with a 10-min incubation for each buffer. The strips were then loaded onto 11% homogeneous acrylamide gels and sealed with 0.5% (w/v) agarose in SDS running buffer (25 mM Tris base, 192 mM glycine, 0.1% (w/v) SDS). The second dimension, the SDS-PAGE, was run at 20°C using a PROTEAN II Multi-Cell (Bio-Rad, USA). The run was first conducted at 16 mA/gel for 30 min followed by 24 mA/gel for 5 h. The gels were stained using the colloidal Coomassie stain method [71] and Brilliant Blue G-250.

### Image analysis

The stained gels were scanned at a 600 dpi (dots per inch) resolution using a PowerLook 2100XL scanner (Umax, USA). Automated detection and matching, quantification and annotation of spots were performed using the PDQuest software package (version 8.0.1, Bio-Rad). The 2-DE was performed with three infected and three uninfected control bird samples, and each sample was repeated 2~3 times. Finally, we selected three representative gels for the control group and three for the infected group, the reproducibility within each group was approximately 85% or better. The gel images were then normalised according to the total quantity in the analysis set. Protein spots (FC≥2) were compared using Student’s t-test and the Statistical Package for the Social Sciences (version 16.0). Spots that had both P≤0.05 and ≥2-fold difference in mean normalised volumes were considered significantly different.

### Protein identification

Differentially expressed protein spots from fresh CCB-stained gels were excised and plated in 96-well microtitre plates. The excised spots were first destained twice with 60 μl of 50 mM NH_4_HCO_3_ and 50% acetonitrile and subsequently dried twice with 60 μl of acetonitrile. The dried pieces of gel were then incubated in ice-cold digestion solution (12.5 ng/μl trypsin and 20 mM NH_4_HCO_3_) for 20 min and transferred into a 37°C incubator for an overnight digestion. Finally, the peptides in the supernatant were collected after being extracted twice with 60 μl of the extraction solution (5% formic acid in 50% acetonitrile).

The peptide solution described above was dried under the protection of N_2_. A 0.8 μl aliquot of matrix solution (5 mg/ml α-cyano-4-hydroxy-cinnamic acid diluted in 0.1% TFA, 50% ACN) was pipetted to dissolve it and subsequently spotted onto a matrix-assisted laser desorption ionisation (MALDI) target plate (Applied Biosystems). The MS analysis of the peptides was performed using an ABI 4700 TOF-TOF Proteomics apparatus (Applied Biosystems). The ultraviolet (UV) laser was operated at a 200 Hz repetition rate with a wavelength of 355 nm. The accelerated voltage was operated at 20 kV, and the mass resolution was maximised at 1500 Da. Myoglobin digested with trypsin was used to calibrate the mass instrument using the internal calibration mode. All the spectra acquired from the samples were processed using 4700 ExploreTM Software (Applied Biosystems) in the default mode. The data were searched using GPS Explorer (v3.6) with the search engine MASCOT(2.1). The search parameters were as follows: the database, NCBInr; taxonomy, Viridiplantae (green plants); the protein molecular mass, from 700 to 4000 Da; trypsin digestion with one missing cleavage; MS tolerance, 100 ppm; and MS/MS tolerance, 0.6 Da. Proteins with scores greater than 71 or a best ion score (MS/MS) of more than 30 were considered to be significant (P<0.05). This work was performed in collaboration with the Proteome Research Center of Fudan University. Typically, ten identities from one spot were provided, and the proteins of *Gallus gallus* (species) and the highest scores were selected according to the criteria for the determination of proteins, as provided by the Proteome Research Center of Fudan University.

### Gene Ontology (GO) annotation using the Agbase database

The proteins were analysed using the AgBase database ( http://www.agbase.msstate.edu). The list of accession numbers was entered into GORetriever to return all existing GO annotations available for that dataset. GORetriever also provides a list of proteins without GO annotation and enters this second list into GOanna to retrieve GO annotations assigned on the basis of sequence similarities. The resulting annotations were summarised on the basis of the GOA whole proteome GOSlim set using GOSlimViewer.

### Protein functional interaction network analysis

The functional interaction networks of the proteins were analysed using STRING ( http://string.embl.de), which is a database of known and predicted protein interactions and includes direct (physical) and indirect (functional) associations. The list of protein names was entered into a table to provide the network of protein–protein interactions. The Occurrence and Coexpression programs provided the functional associations of the proteins. Functionally associated proteins often have similar phylogenetic profiles and/or display the phenomenon of co-expression.

### Real-time PCR

The expression levels of ANXA1, MIF, OP18, VIM and NOM1 in the thymus of the chickens were determined using real-time PCR (7500 Real-Time PCR System, ABI). The sequences of the primers are provided in Table 2, and the primer sequences for MIF, GAPDH and Meq have been previously reported [72,73]. The primers were synthesised by Shenergy Biocolor Bioscience & Technology Company (Shanghai, China). Total RNA was prepared from ground thymus tissue using the AxyPrep^™^ Multisource Total RNA Miniprep kit (AXYGEN, USA). Total RNA (1 μg) was reverse transcribed into first-strand cDNA using the PrimeScript RT Master Mix (TaKaRa, USA) following the manufacturer’s instructions, and the synthesised cDNA was diluted 1:10 with nuclease-free water. Diluted cDNA (1 μl), 400 nM primers and 10 μl of SYBR Green Master Mix were used for the real-time PCR in a final volume of 20 μl. The amplification conditions were: 95°C for 30 s, followed by 40 cycles of 95°C for 5 s, and 60°C for 34 s. Dissociation curves were generated to analyse the individual PCR products after 40 cycles. The expression levels of five proteins were normalised against the expression of chicken glyceraldehyde-3-phosphate dehydrogenase (GAPDH) mRNA. The analyses of the data for relative gene expression were performed using the 2 ^–△△CT^ method [74].

**Table 2:** Primers used for real-time PCR

**Gene Symbol**	**Nucleotide sequence**	**Product size (bp)**	**Accession number**
**MIF**	**F 5**^′^**-GCCCGCGCAGTACATAGC-3**^′^	**57**	**XM42_5824**
	**R 5**^′^**-CCCCCGAAGGACATCATCT-3**^′^		
**OP18**	**F 5**^′^**-TTGAGCTGATCCTTGGTCCC-3**^′^	**128**	**NM_001001858.1**
	**R 5**^′^**-CTTGCGTCTCTCTTCTGC-3**^′^		
**VIM**	**F 5**^′^**-CAACACGGAGTTCAAGGCGA-3**^′^	**79**	**NM_001048076.1**
	**R 5**^′^**-GATGTAGTTGGCGAAGCGGT-3**^′^		
**NPM1**	**F 5**^′^**-GGTTACATTAGGGGCTGG-3**^′^	**73**	**NM_205267.1**
	**R 5**^′^**-GTTGCCTTCGTAGTCCAGTG-3**^′^		
**ANXA1**	**F 5**^′^**-AAAACTGCCTGACTGCCCTT-3**^′^	**90**	**NM_206906.1**
	**R 5**^′^**-TTCCACTCCCCTTCATTGCC-3**^′^		
**Meq**	**F 5**^′^**-GTCCCCCCTCGATCTTTCTC −3**^′^	**184**	**NC_002229.3**
	**R 5**^′^**-CGTCTGCTTCCTGCGTCTTC-3**^′^		
**gB**	**F 5**^′^**-ACCCCATTCGGTGGCTTTTC-3**^′^	**122**	**NC_002229.3**
	**R 5**^′^**-GCGTCCAGTTGTCTGAGG-3**^′^		
**GAPDH**	**F 5**^′^**-AGGGTGGTGCTAAGCGTGTTA-3**^′^	**78**	**NM_204305**
	**R 5**^′^**-TCTCATGGTTGACACCCATCA-3**^′^		

## Competing interests

The authors declare that they have no competing interests.

## Authors’ contributions

AQ supervised all the experiments and revised the manuscript. XH performed the experiments and prepared the manuscript. HX, WX and JM assistant research. KQ. provided a critical review of the manuscript. CY helped to conduct the real-time PCR for MIF. All the authors read and approved the final manuscript.

## References

[B1] Witter RL Schat KA Marek’s disease 2003 Ames, Iowa: Iowa state University Press

[B2] Davison F Nair V Use of Marek’s disease vaccines: could they be driving the virus to increasing virulence? Expert Rev Vaccines 2005 4 77 88 10.1586/14760584.4.1.77 15757475

[B3] Osterrieder N Kamil JP Schumacher D Tischer BK Trapp S Marek’s disease virus: from miasma to model Nat Rev Microbiol 2006 4 283 294 10.1038/nrmicro1382 16541136

[B4] Jarosinski KW Tischer BK Trapp S Osterrieder N Marek’s disease virus: lytic replication, oncogenesis and control Expert Rev Vaccines 2006 5 761 772 10.1586/14760584.5.6.761 17184215

[B5] Thanthrige-Don N Abdul-Careem MF Shack LA Burgess SC Sharif S Analyses of the spleen proteome of chickens infected with Marek’s disease virus Virology 2009 390 356 367 10.1016/j.virol.2009.05.020 19540544 PMC7103390

[B6] Lu Z Qin A Qian K Chen X Jin W Zhu Y Eltahir YM Proteomic analysis of the host response in the bursa of Fabricius of chickens infected with Marek’s disease virus Virus Res 2010 153 250 257 10.1016/j.virusres.2010.08.010 20723570

[B7] Thanthrige-Don N Parvizi P Sarson AJ Shack LA Burgess SC Sharif S Proteomic analysis of host responses to Marek’s disease virus infection in spleens of genetically resistant and susceptible chickens Dev Comp Immunol 2010 34 699 704 10.1016/j.dci.2010.01.016 20138080

[B8] Niikura M Kim T Hunt HD Burnside J Morgan RW Dodgson JB Cheng HH Marek’s disease virus up-regulates major histocompatibility complex class II cell surface expression in infected cells Virology 2007 359 212 219 10.1016/j.virol.2006.09.010 17028059

[B9] Thanthrige-Don N Read LR Abdul-Careem MF Mohammadi H Mallick AI Sharif S Marek’s disease virus influences the expression of genes associated with IFN-gamma-inducible MHC class II expression Viral Immunol 2010 23 227 232 10.1089/vim.2009.0092 20374003

[B10] Morimura T Hattori M Ohashi K Sugimoto C Onuma M Immunomodulation of peripheral T cells in chickens infected with Marek’s disease virus: involvement in immunosuppression J Gen Virol 1995 76 Pt 12 2979 2985 8847503 10.1099/0022-1317-76-12-2979

[B11] Morimura T Ohashi K Kon Y Hattori M Sugimoto C Onuma M Apoptosis and CD8-down-regulation in the thymus of chickens infected with Marek’s disease virus Arch Virol 1996 141 2243 2249 10.1007/BF01718230 8973538

[B12] Morimura T Ohashi K Kon Y Hattori M Sugimoto C Onuma M Apoptosis in peripheral CD4+T cells and thymocytes by Marek’s disease virus-infection Leukemia 1997 11 Suppl 3 206 208 9209342

[B13] Sharma JM Witter RL Burmester BR Pathogenesis of Marek’s disease in old chickens: lesion regression as the basis for age-related resistance Infect Immun 1973 8 715 724 4584047 10.1128/iai.8.5.715-724.1973 PMC422917

[B14] Deng X Li X Shen Y Qiu Y Shi Z Shao D Jin Y Chen H Ding C Li L The Meq oncoprotein of Marek’s disease virus interacts with p53 and inhibits its transcriptional and apoptotic activities Virol J 2010 7 348 10.1186/1743-422X-7-348 21110861 PMC2999606

[B15] Gimeno IM Witter RL Hunt HD Lee LF Reddy SM Neumann U Marek’s disease virus infection in the brain: virus replication, cellular infiltration, and major histocompatibility complex antigen expression Vet Pathol 2001 38 491 503 10.1354/vp.38-5-491 11572556

[B16] Barrow AD Burgess SC Baigent SJ Howes K Nair VK Infection of macrophages by a lymphotropic herpesvirus: a new tropism for Marek’s disease virus J Gen Virol 2003 84 2635 2645 10.1099/vir.0.19206-0 13679597

[B17] Frascaroli G Varani S Blankenhorn N Pretsch R Bacher M Leng L Bucala R Landini MP Mertens T Human cytomegalovirus paralyzes macrophage motility through down-regulation of chemokine receptors, reorganization of the cytoskeleton, and release of macrophage migration inhibitory factor J Immunol 2009 182 477 488 19109179 10.4049/jimmunol.182.1.477 PMC3618717

[B18] Regis EG Barreto-de-Souza V Morgado MG Bozza MT Leng L Bucala R Bou-Habib DC Elevated levels of macrophage migration inhibitory factor (MIF) in the plasma of HIV-1-infected patients and in HIV-1-infected cell cultures: a relevant role on viral replication Virology 2010 399 31 38 10.1016/j.virol.2009.12.018 20085845 PMC3140709

[B19] Backes P Quinkert D Reiss S Binder M Zayas M Rescher U Gerke V Bartenschlager R Lohmann V Role of annexin A2 in the production of infectious hepatitis C virus particles J Virol 2010 84 5775 5789 10.1128/JVI.02343-09 20335258 PMC2876593

[B20] Szebeni A Mehrotra B Baumann A Adam SA Wingfield PT Olson MO Nucleolar protein B23 stimulates nuclear import of the HIV-1 Rev protein and NLS-conjugated albumin Biochemistry 1997 36 3941 3949 10.1021/bi9627931 9092824

[B21] Li YP Protein B23 is an important human factor for the nucleolar localization of the human immunodeficiency virus protein Tat J Virol 1997 71 4098 4102 9094689 10.1128/jvi.71.5.4098-4102.1997 PMC191564

[B22] Burch AD Weller SK Herpes simplex virus type 1 DNA polymerase requires the mammalian chaperone hsp90 for proper localization to the nucleus J Virol 2005 79 10740 10749 10.1128/JVI.79.16.10740-10749.2005 16051866 PMC1182622

[B23] Sun X Barlow EA Ma S Hagemeier SR Duellman SJ Burgess RR Tellam J Khanna R Kenney SC Hsp90 inhibitors block outgrowth of EBV-infected malignant cells in vitro and in vivo through an EBNA1-dependent mechanism Proc Natl Acad Sci U S A 2010 107 3146 3151 10.1073/pnas.0910717107 20133771 PMC2840277

[B24] Wen KW Damania B Hsp90 and Hsp40/Erdj3 are required for the expression and anti-apoptotic function of KSHV K1 Oncogene 2010 29 3532 3544 10.1038/onc.2010.124 20418907 PMC2908282

[B25] Bacher M Metz CN Calandra T Mayer K Chesney J Lohoff M Gemsa D Donnelly T Bucala R An essential regulatory role for macrophage migration inhibitory factor in T-cell activation Proc Natl Acad Sci U S A 1996 93 7849 7854 10.1073/pnas.93.15.7849 8755565 PMC38837

[B26] Calandra T Roger T Macrophage migration inhibitory factor: a regulator of innate immunity Nat Rev Immunol 2003 3 791 800 10.1038/nri1200 14502271 PMC7097468

[B27] Flaster H Bernhagen J Calandra T Bucala R The macrophage migration inhibitory factor-glucocorticoid dyad: regulation of inflammation and immunity Mol Endocrinol 2007 21 1267 1280 10.1210/me.2007-0065 17389748

[B28] Roger T Chanson AL Knaup-Reymond M Calandra T Macrophage migration inhibitory factor promotes innate immune responses by suppressing glucocorticoid-induced expression of mitogen-activated protein kinase phosphatase-1 Eur J Immunol 2005 35 3405 3413 10.1002/eji.200535413 16224818

[B29] Aeberli D Yang Y Mansell A Santos L Leech M Morand EF Endogenous macrophage migration inhibitory factor modulates glucocorticoid sensitivity in macrophages via effects on MAP kinase phosphatase-1 and p38 MAP kinase FEBS Lett 2006 580 974 981 10.1016/j.febslet.2006.01.027 16442105

[B30] Toh ML Aeberli D Lacey D Yang Y Santos LL Clarkson M Sharma L Clyne C Morand EF Regulation of IL-1 and TNF receptor expression and function by endogenous macrophage migration inhibitory factor J Immunol 2006 177 4818 4825 16982923 10.4049/jimmunol.177.7.4818

[B31] D’Acquisto F Perretti M Flower RJ Annexin-A1: a pivotal regulator of the innate and adaptive immune systems Br J Pharmacol 2008 155 152 169 18641677 10.1038/bjp.2008.252 PMC2538690

[B32] D’Acquisto F On the adaptive nature of annexin-A1 Curr Opin Pharmacol 2009 9 521 528 10.1016/j.coph.2009.04.007 19481503

[B33] Srivastava P Interaction of heat shock proteins with peptides and antigen presenting cells: chaperoning of the innate and adaptive immune responses Annu Rev Immunol 2002 20 395 425 10.1146/annurev.immunol.20.100301.064801 11861608

[B34] Javid B MacAry PA Lehner PJ Structure and function: heat shock proteins and adaptive immunity J Immunol 2007 179 2035 2040 17675458 10.4049/jimmunol.179.4.2035

[B35] Kunisawa J Shastri N Hsp90alpha chaperones large C-terminally extended proteolytic intermediates in the MHC class I antigen processing pathway Immunity 2006 24 523 534 10.1016/j.immuni.2006.03.015 16713971

[B36] Levy AM Davidson I Burgess SC Dan Heller E Major histocompatibility complex class I is downregulated in Marek’s disease virus infected chicken embryo fibroblasts and corrected by chicken interferon Comp Immunol Microbiol Infect Dis 2003 26 189 198 10.1016/S0147-9571(02)00055-3 12581748

[B37] Jarosinski KW Hunt HD Osterrieder N Down-regulation of MHC class I by the Marek’s disease virus (MDV) UL49.5 gene product mildly affects virulence in a haplotype-specific fashion Virology 2010 405 457 463 10.1016/j.virol.2010.06.041 20637486

[B38] Mitchell RA Liao H Chesney J Fingerle-Rowson G Baugh J David J Bucala R Macrophage migration inhibitory factor (MIF) sustains macrophage proinflammatory function by inhibiting p53: regulatory role in the innate immune response Proc Natl Acad Sci U S A 2002 99 345 350 10.1073/pnas.012511599 11756671 PMC117563

[B39] Salminen A Kaarniranta K Control of p53 and NF-kappaB signaling by WIP1 and MIF: role in cellular senescence and organismal aging Cell Signal 2011 23 747 752 10.1016/j.cellsig.2010.10.012 20940041

[B40] Venugopal K Payne LN Molecular pathogenesis of Marek’s disease-recent developments Avian Pathol 1995 24 597 609 10.1080/03079459508419100 18645817

[B41] Johnsen JI Aurelio ON Kwaja Z Jorgensen GE Pellegata NS Plattner R Stanbridge EJ Cajot JF p53-mediated negative regulation of stathmin/Op18 expression is associated with G(2)/M cell-cycle arrest Int J Cancer 2000 88 685 691 10.1002/1097-0215(20001201)88:5<685::AID-IJC1>3.0.CO;2-Z 11072234

[B42] Fang L Min L Lin Y Ping G Rui W Ying Z Xi W Ting H Li L Ke D Downregulation of stathmin expression is mediated directly by Egr1 and associated with p53 activity in lung cancer cell line A549 Cell Signal 2010 22 166 173 10.1016/j.cellsig.2009.09.030 19786090

[B43] Arur S Uche UE Rezaul K Fong M Scranton V Cowan AE Mohler W Han DK Annexin I is an endogenous ligand that mediates apoptotic cell engulfment Dev Cell 2003 4 587 598 10.1016/S1534-5807(03)00090-X 12689596

[B44] Sakaguchi M Murata H Sonegawa H Sakaguchi Y Futami J Kitazoe M Yamada H Huh NH Truncation of annexin A1 is a regulatory lever for linking epidermal growth factor signaling with cytosolic phospholipase A2 in normal and malignant squamous epithelial cells J Biol Chem 2007 282 35679 35686 10.1074/jbc.M707538200 17932043

[B45] Solito E de Coupade C Canaider S Goulding NJ Perretti M Transfection of annexin 1 in monocytic cells produces a high degree of spontaneous and stimulated apoptosis associated with caspase-3 activation Br J Pharmacol 2001 133 217 228 10.1038/sj.bjp.0704054 11350857 PMC1572776

[B46] Debret R El Btaouri H Duca L Rahman I Radke S Haye B Sallenave JM Antonicelli F Annexin A1 processing is associated with caspase-dependent apoptosis in BZR cells FEBS Lett 2003 546 195 202 10.1016/S0014-5793(03)00570-2 12832039

[B47] Tabe Y Jin L Contractor R Gold D Ruvolo P Radke S Xu Y Tsutusmi-Ishii Y Miyake K Miyake N Novel role of HDAC inhibitors in AML1/ETO AML cells: activation of apoptosis and phagocytosis through induction of annexin A1 Cell Death Differ 2007 14 1443 1456 10.1038/sj.cdd.4402139 17464329

[B48] Mitchell RA Bucala R Tumor growth-promoting properties of macrophage migration inhibitory factor (MIF) Semin Cancer Biol 2000 10 359 366 10.1006/scbi.2000.0328 11100884

[B49] Bifulco C McDaniel K Leng L Bucala R Tumor growth-promoting properties of macrophage migration inhibitory factor Curr Pharm Des 2008 14 3790 3801 10.2174/138161208786898608 19128232

[B50] Conroy H Mawhinney L Donnelly SC Inflammation and cancer: macrophage migration inhibitory factor (MIF)–the potential missing link QJM 2010 103 831 836 10.1093/qjmed/hcq148 20805118 PMC2955282

[B51] Rendon BE Willer SS Zundel W Mitchell RA Mechanisms of macrophage migration inhibitory factor (MIF)-dependent tumor microenvironmental adaptation Exp Mol Pathol 2009 86 180 185 10.1016/j.yexmp.2009.01.001 19186177 PMC2680445

[B52] Mussunoor S Murray GI The role of annexins in tumour development and progression J Pathol 2008 216 131 140 10.1002/path.2400 18698663

[B53] Zhu F Xu C Jiang Z Jin M Wang L Zeng S Teng L Cao J Nuclear localization of annexin A1 correlates with advanced disease and peritoneal dissemination in patients with gastric carcinoma Anat Rec (Hoboken) 2010 293 1310 1314 10.1002/ar.21176 20665809

[B54] Rondepierre F Bouchon B Papon J Bonnet-Duquennoy M Kintossou R Moins N Maublant J Madelmont JC D’Incan M Degoul F Proteomic studies of B16 lines: involvement of annexin A1 in melanoma dissemination Biochim Biophys Acta 2009 1794 61 69 10.1016/j.bbapap.2008.09.014 18952200

[B55] de Graauw M van Miltenburg MH Schmidt MK Pont C Lalai R Kartopawiro J Pardali E Le Devedec SE Smit VT van der Wal A Annexin A1 regulates TGF-beta signaling and promotes metastasis formation of basal-like breast cancer cells Proc Natl Acad Sci U S A 2010 107 6340 6345 10.1073/pnas.0913360107 20308542 PMC2852023

[B56] Su N Xu XY Chen H Gao WC Ruan CP Wang Q Sun YP Increased expression of annexin A1 is correlated with K-ras mutation in colorectal cancer Tohoku J Exp Med 2010 222 243 250 10.1620/tjem.222.243 21127395

[B57] Yi M Schnitzer JE Impaired tumor growth, metastasis, angiogenesis and wound healing in annexin A1-null mice Proc Natl Acad Sci U S A 2009 106 17886 17891 10.1073/pnas.0901324106 19805119 PMC2764877

[B58] Ivaska J Pallari HM Nevo J Eriksson JE Novel functions of vimentin in cell adhesion, migration, and signaling Exp Cell Res 2007 313 2050 2062 10.1016/j.yexcr.2007.03.040 17512929

[B59] Dutsch-Wicherek M RCAS1, MT, and vimentin as potential markers of tumor microenvironment remodeling Am J Reprod Immunol 2010 63 181 188 10.1111/j.1600-0897.2009.00803.x 20085563

[B60] Bunnell TM Burbach BJ Shimizu Y Ervasti JM beta-Actin specifically controls cell growth, migration and the G-actin pool Mol Biol Cell 2011 22 4047 4058 10.1091/mbc.E11-06-0582 21900491 PMC3204067

[B61] Popow A Nowak D Malicka-Blaszkiewicz M Actin cytoskeleton and beta-actin expression in correlation with higher invasiveness of selected hepatoma Morris 5123 cells J Physiol Pharmacol 2006 57 Suppl 7 111 123 17228099

[B62] Okuwaki M The structure and functions of NPM1/Nucleophsmin/B23, a multifunctional nucleolar acidic protein J Biochem 2008 143 441 448 18024471 10.1093/jb/mvm222

[B63] Zhao Y Kurian D Xu H Petherbridge L Smith LP Hunt L Nair V Interaction of Marek’s disease virus oncoprotein Meq with heat-shock protein 70 in lymphoid tumour cells J Gen Virol 2009 90 2201 2208 10.1099/vir.0.012062-0 19494050

[B64] Russell SE Hall PA Do septins have a role in cancer? Br J Cancer 2005 93 499 503 10.1038/sj.bjc.6602753 16136025 PMC2361591

[B65] Roeseler S Sandrock K Bartsch I Zieger B Septins, a novel group of GTP-binding proteins: relevance in hemostasis, neuropathology and oncogenesis Klin Padiatr 2009 221 150 155 10.1055/s-0029-1220706 19437362

[B66] Amir S Wang R Simons JW Mabjeesh NJ SEPT9_v1 up-regulates hypoxia-inducible factor 1 by preventing its RACK1-mediated degradation J Biol Chem 2009 284 11142 11151 19251694 10.1074/jbc.M808348200 PMC2670119

[B67] Gonzalez ME Makarova O Peterson EA Privette LM Petty EM Up-regulation of SEPT9_v1 stabilizes c-Jun-N-terminal kinase and contributes to its pro-proliferative activity in mammary epithelial cells Cell Signal 2009 21 477 487 10.1016/j.cellsig.2008.11.007 19071215 PMC2811713

[B68] Carpenter B MacKay C Alnabulsi A MacKay M Telfer C Melvin WT Murray GI The roles of heterogeneous nuclear ribonucleoproteins in tumour development and progression Biochim Biophys Acta 2006 1765 85 100 16378690 10.1016/j.bbcan.2005.10.002

[B69] Ford LP Wright WE Shay JW A model for heterogeneous nuclear ribonucleoproteins in telomere and telomerase regulation Oncogene 2002 21 580 583 10.1038/sj.onc.1205086 11850782

[B70] Bradford MM A rapid and sensitive method for the quantitation of microgram quantities of protein utilizing the principle of protein-dye binding Anal Biochem 1976 72 248 254 10.1016/0003-2697(76)90527-3 942051

[B71] Candiano G Bruschi M Musante L Santucci L Ghiggeri GM Carnemolla B Orecchia P Zardi L Righetti PG Blue silver: a very sensitive colloidal Coomassie G-250 staining for proteome analysis Electrophoresis 2004 25 1327 1333 10.1002/elps.200305844 15174055

[B72] Kim S Miska KB Jenkins MC Fetterer RH Cox CM Stuard LH Dalloul RA Molecular cloning and functional characterization of the avian macrophage migration inhibitory factor (MIF) Dev Comp Immunol 2010 34 1021 1032 10.1016/j.dci.2010.05.005 20470818

[B73] Abdul-Careem MF Hunter BD Nagy E Read LR Sanei B Spencer JL Sharif S Development of a real-time PCR assay using SYBR Green chemistry for monitoring Marek’s disease virus genome load in feather tips J Virol Methods 2006 133 34 40 10.1016/j.jviromet.2005.10.018 16300836

[B74] Livak KJ Schmittgen TD Analysis of relative gene expression data using real-time quantitative PCR and the 2(−Delta Delta C(T)) Method Methods 2001 25 402 408 10.1006/meth.2001.1262 11846609

